# Genital tuberculosis initially presenting as pyometra and progressing to miliary tuberculosis: A case report

**DOI:** 10.1016/j.rmcr.2026.102376

**Published:** 2026-01-20

**Authors:** Kentaro Kasuga, Miyako Kitazono, Masaomi Maeda, Taro Koba, Shuichi Matsuda, Mikio Takamori

**Affiliations:** Department of Respiratory Medicine and Medical Oncology, Tokyo Metropolitan Tama Medical Center, 2-8-29 Musashidai, Fuchu, Tokyo, 183-8524, Japan

**Keywords:** Genital tuberculosis, Pyometra, Miliary tuberculosis

## Abstract

Female genital tuberculosis is a rare and often asymptomatic condition, which makes its diagnosis challenging. Early recognition of uncommon clinical presentation of tuberculosis is crucial for the better management of it. We report herein a case of genital tuberculosis that initially presented as pyometra and subsequently progressed to miliary tuberculosis. An 86-year-old woman with a history of immunosuppressive therapy for bullous pemphigoid presented with fever and septic shock and was diagnosed with pyelonephritis. Computed tomography (CT) incidentally revealed pyometra, from which *Escherichia coli* was isolated, and showed no abnormal findings in the lung fields. At the time, an acid-fast bacillus and histopathological examination had not been conducted. Although she improved with antibiotics, her fever recurred one month later. Repeat CT demonstrated diffuse, granular opacities in the bilateral lungs and an enlargement of the pyometra. Eventually, she was diagnosed with genital tuberculosis co-occurring with miliary tuberculosis. Both the pyometra and miliary tuberculosis improved with anti-tuberculosis treatment. Given the course of pyometra preceding miliary tuberculosis, we speculate that the progression of the tuberculous pyometra caused the disseminated disease. This case emphasizes that delays in the diagnosis of tuberculosis lead to severe disease. *Mycobacterium tuberculosis* should be considered a potential causative pathogen of pyometra.

## Highlights

• Genital tuberculosis is rare in low tuberculosis burden countries.
• Pyometra is more common in frail elderly post-menopausal women.
• Tuberculosis should be considered as a possible cause of pyometra.

## 1 Introduction

Genital tuberculosis is rare and often asymptomatic, and its diagnosis is challenging [[1], [2], [3], [4]]. Female genital tuberculosis reportedly occurs in 0.002–0.56 % of hospitalized women and in 0.2–21 % of infertile women, although the prevalence varies significantly by country and region due to differences in tuberculosis burden [3,[5], [6], [7], [8]]. The patients with genital tuberculosis have been reported to be approximately 0.1 % in all female patients with active tuberculosis in Japan, a low tuberculosis prevalence country [9]. In genital tuberculosis, *M. tuberculosis* is thought to be primarily transmitted hematogenously from the lungs to the fallopian tubes and endometrium during the primary infection [[10], [11], [12], [13]]. Most cases of genital tuberculosis are relapses stemming from a latent site years or even decades after the initial infection [4,13].

Pyometra occurs in 0.5–2.5 % of uterine tuberculosis cases [14]. The development of pyometra is attributed to impaired uterine clearance in elderly post-menopausal women with functional decline, and the symptoms are often non-specific [15]. Aerobic and anaerobic organisms account for almost all the causative pathogens in pyometra [15], while cases due to *Mycobacterium tuberculosis (M. tuberculosis)* are rare. Diagnosis of tuberculous pyometra requires microbiologic and histopathologic evaluation of specimens collected from endometrium, including nucleic acid amplification tests [16]. Low incidence rate and insidious disease progression reduce the opportunity of clinical suspicion and delay the diagnosis of tuberculous pyometra.

We report herein a case of genital tuberculosis that initially presented as pyometra, then progressed to miliary tuberculosis.

This case report has been reported in line with the 2025 SCARE criteria.

## 2 Case presentation

**Fig. 1:**
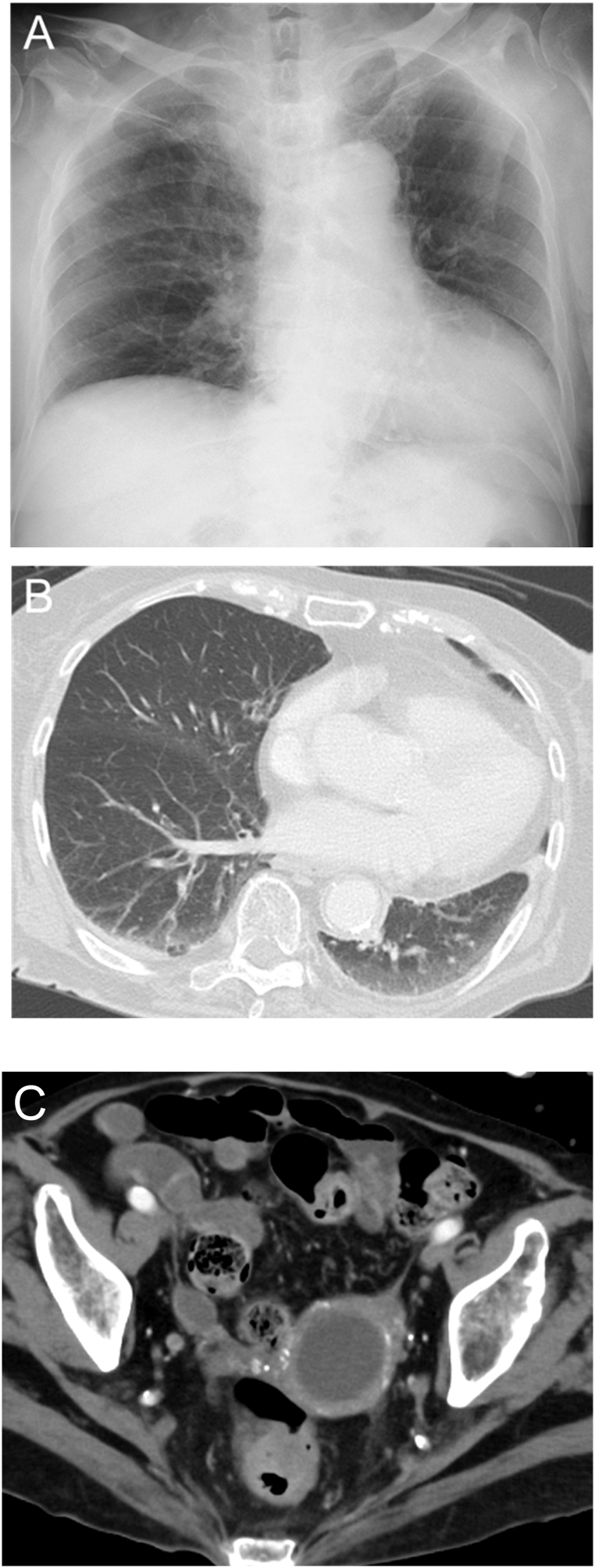
CT findings at one month before admission. A. Chest CT demonstrated no evident abnormalities in the lung fields. B. Abdominal and pelvic CT demonstrated the uterine pyometra.

**Fig. 2:**
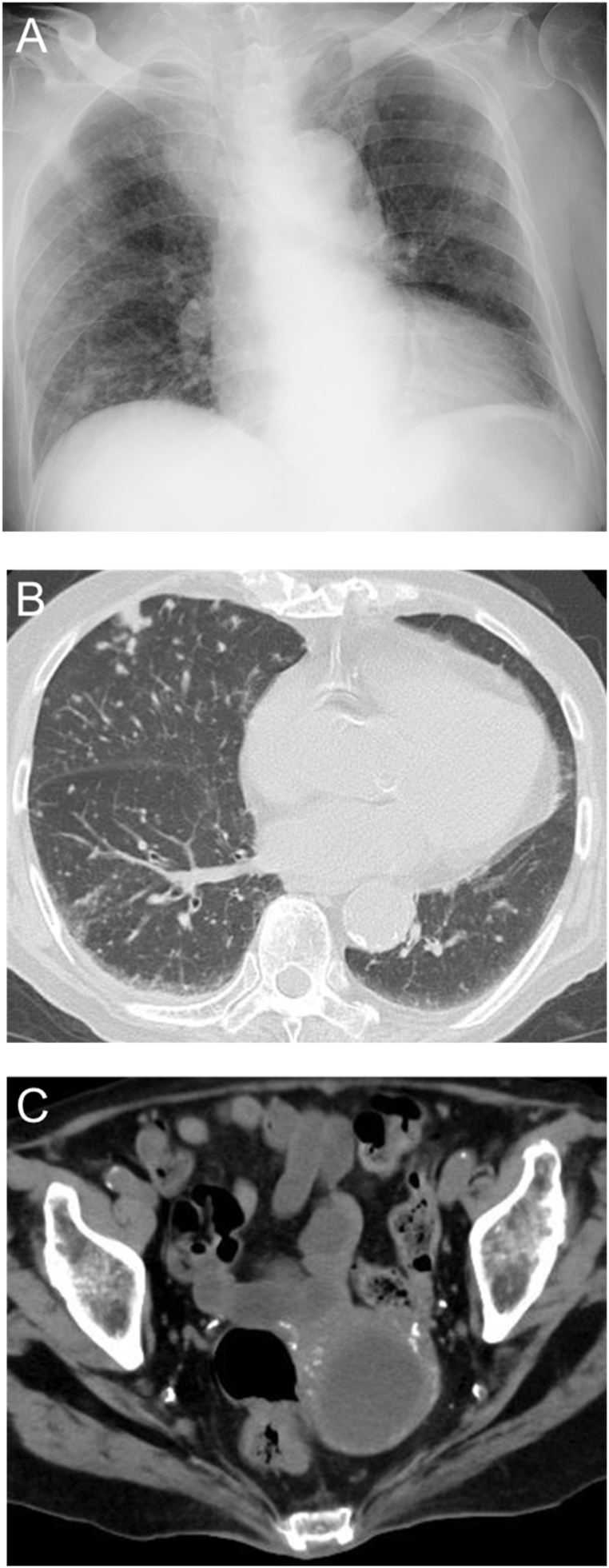
CT findings on admission to our hospital. A. Chest CT demonstrated diffuse, bilateral, granular opacities. B. Abdominal and pelvic CT demonstrated an enlargement of the uterine pyometra.

Four months before referred to our hospital, an 86-year-old woman with diabetes mellitus was diagnosed with bullous pemphigoid, began immunosuppressive therapy with prednisolone 20 mg/day, cyclosporine 50 mg/day, and intravenous immunoglobulin 20,000 mg/day (400 mg/kg/day). Three months later, she was admitted to a previous hospital with a fever and septic shock, with diagnosis of pyelonephritis. Computed tomography (CT) incidentally revealed pyometra, but no evident abnormalities in the lung fields (Fig. 1). Endometrial aspiration was performed, and *Escherichia coli (E. coli)* was isolated from a general bacterial culture of the uterine contents, while an acid-fast bacillus (AFB) culture was not obtained and a histopathological examination was not performed due to a negative cytology result. Ampicillin/sulbactam was administered, with cessation of cyclosporin. She recovered from septic shock by glucocorticoid replacement therapy considering relative adrenal insufficiency. However, one month later, her fever recurred. Chest X-ray and CT revealed diffuse, granular opacities in the bilateral lungs and an enlargement of the pyometra (Fig. 2). Although both the AFB smear and nucleic acid amplification test (NAAT) for *M. tuberculosis* were negative in the sputum, the uterine contents tested positive on both tests. She was transferred to our hospital for quarantine, further evaluation, and treatment.

She was diagnosed with progressive supranuclear palsy during the previous hospitalization, and was bedridden and fully dependent in all activities of daily living. Her medication included prednisolone 15 mg/day, trimethoprim 40 mg/day, sulfamethoxazole 200 mg/day, rabeprazole 10 mg/day, levodopa 300 mg/day, and carbidopa 30 mg/day on admission. She had no history of infertility (gravida 4, para 4) and no family history of tuberculosis. Physical examination revealed a Glasgow Coma Scale score of 14 (E4V4M6), body temperature of 37.2 °C, blood pressure of 102/80 mmHg, heart rate of 86 beats/min, and SpO_2_ of 94 % on room air. Chest and abdominal examinations were unremarkable. Blood tests revealed elevated white blood cell count and C-reactive protein level, and positive result in the T-SPOT test (Table 1). The AFB smears of the sputum and uterine contents were ± and 2+, respectively, and NAATs for *M. tuberculosis* were positive in both specimens. AFB cultures identified *M. tuberculosis* in the sputum and uterine contents, but not in the blood or urine. General bacterial culture of the uterine contents yielded *E. coli* and *Klebsiella oxytoca* (*K. oxytoca*). Cytological examination of the uterine contents revealed no malignant cells. She was diagnosed with genital tuberculosis and miliary tuberculosis. Anti-tuberculosis therapy was initiated with isoniazid 250 mg/day, rifampicin 450 mg/day, ethambutol 750 mg/day, and pyrazinamide 1100 mg/day. Drug susceptibility testing confirmed sensitivity to all anti-tuberculosis drugs.

**Table 1:** Laboratory findings performed on admission to our hospital.

Hematology			Biochemistry and serology		
White blood cells	8000	/μL	Total protein	6.1	g/dL
Neutrophils	82	%	Albumin	2.5	g/dL
Lymphocytes	11	%	Total bilirubin	1.1	mg/dL
Monocytes	5	%	AST	16	IU/L
Eosinophils	1	%	ALT	10	IU/L
Hemoglobin	9.1	g/dL	LDH	232	IU/L
Platelet	21.7	x10^4^ μL	Creatine kinase	18	IU/L
ESR (1h)	97	mm	Blood urea nitrogen	13.7	mg/dL
			Creatinine	0.55	mg/dL
			Glucose	221	mg/dL
			Hemoglobin A1c	7.5	%
			C-reactive protein	5.06	mg/dL
					
			IGRA(T-SPOT®)	positive	
			Panel A	35	
			Panel B: 46	46	
			Nil Control	0	
			Positive Control	501	

Abbreviations: ESR, erythrocyte sedimentation rate; AST, aspartate transaminase; ALT, alanine aminotransferase; LDH, lactate dehydrogenase; IGRA, Interferon-γ release assay.

**Fig. 3:**
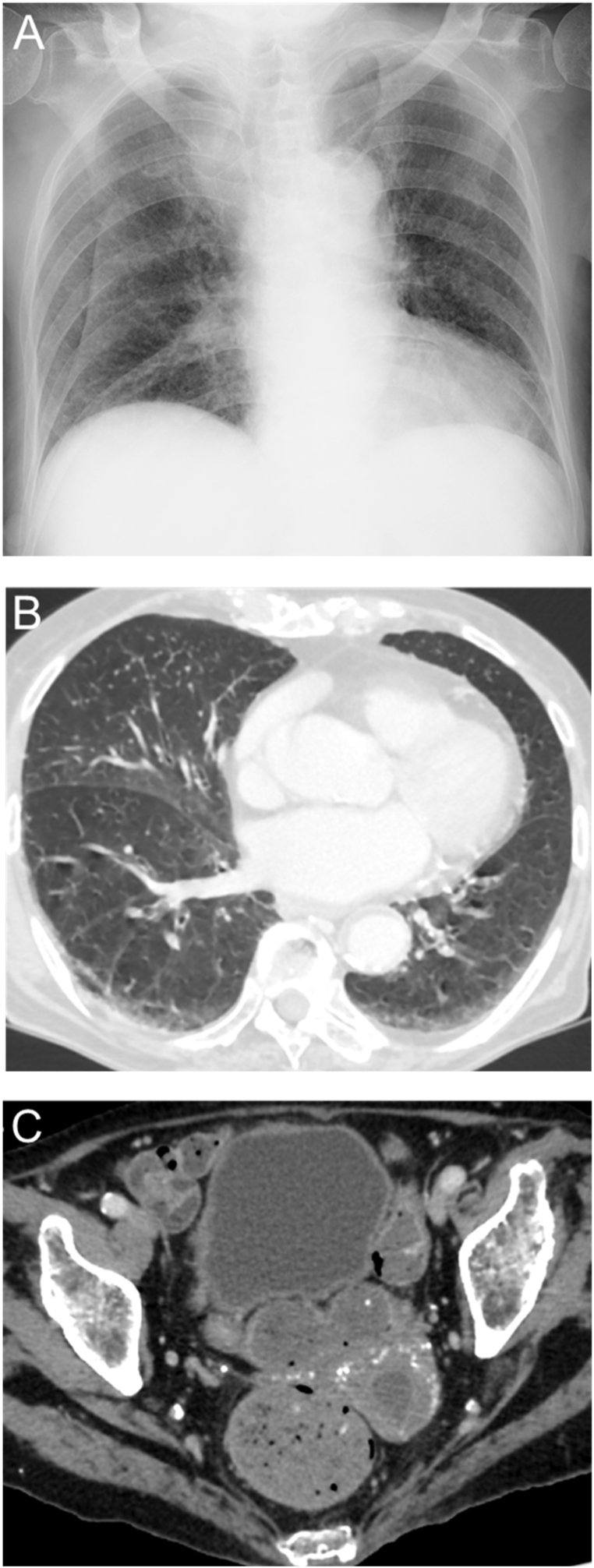
CT findings at one month after anti-tuberculosis treatment. A. Chest CT demonstrated an improvement in the diffuse, bilateral, granular opacities. B. Abdominal and pelvic CT demonstrated a reduction in the size of the pyometra.

One month after the initiation of this treatment, follow-up chest X-ray and CT demonstrated improvement in the bilateral, granular opacities and a significant reduction in the size of the pyometra (Fig. 3). After confirming that three consecutive sputum AFB smears were negative, she was transferred back to the previous hospital on the 35th day of hospitalization.

## 3 Discussion

This case report highlights the difficulty in diagnosing tuberculous pyometra. The progression to miliary tuberculosis provided the opportunity to diagnose the tuberculous pyometra, suggesting that delay in diagnosis of tuberculous pyometra has potential for deterioration. Previous reports of postmenopausal tuberculous pyometra showed favorable outcomes without progression to miliary tuberculosis [2,14,17,18], while our patient fortunately followed an uneventful course after stating anti-tuberculous treatment. Reactivation of tuberculosis in the elderly or immunocompromised hosts resulting from latent infection have become more common in low tuberculosis burden countries [19]. Although tuberculosis is a rare cause of pyometra, understanding atypical presentations of tuberculosis is vital for early detection, timely treatment, and infection control. Our case demonstrates that early suspicion of tuberculosis in the patient with pyometra warrants investigation for active infection through AFB and histopathological examination.

In our case, the patient was diagnosed with tuberculosis following immunosuppressive therapy for bullous pemphigoid and sepsis. Corticosteroid treatment has been associated with an increased risk of tuberculosis [20], and the prior use of other immunosuppressive agents may have contributed to the development of the disease. In addition, it has been reported that immunosuppression during sepsis and prolonged sepsis-induced immunoparalysis can occur, particularly in elderly individuals [21]. Based on these reports, uterine tuberculosis in this patient was most likely a reactivation of latent infection triggered by immunosuppression, resulting from both the initiation of immunosuppressive therapy and the onset of septic shock. We assume that miliary tuberculosis developed secondary to genital tuberculosis, as pyometra preceded the detection of abnormal findings in the lung fields. Previous reports have also described cases in which genital tuberculosis preceded miliary tuberculosis [22,23], supporting the possibility that genital tuberculosis in our patient progressed to miliary tuberculosis. *E. coli* and *K. oxytoca* were also isolated from the uterine contents, and co-infections involving *M. tuberculosis* and other bacteria are not uncommon [24,25]. Although such co-infection may have contributed to the formation of an abscess or localized tissue damage, in our case, the size of the pyometra decreased with anti-tuberculosis treatment alone, without additional antibacterial agents, which implies that the co-infection may not have played a major role.

Tuberculosis remains a major public health concern, particularly among elderly and immunocompromised individuals, who are at increased risk of both disease and death. In parts of the Western Pacific, where the aging population is growing rapidly, elderly individuals account for a large proportion of cases, and tuberculosis remains a leading cause of infectious morbidity and mortality [[26], [27], [28], [29]]. Given the global aging population and the increasing use of immunosuppressive therapies, it is quite possible that similar, clinical cases will be seen in the future. All medical professionals, including obstetricians and gynecologists, should consider *M. tuberculosis* a potential causative pathogen of pyometra and perform a thorough examination, including tests for genital tuberculosis, particularly in elderly or immunosuppressed patients, as well as in cases of antibiotic-resistant pyometra. Even if other bacteria are identified, as in our case, the possibility of *M. tuberculosis* should not be excluded.

All current reports of genital tuberculosis presenting as pyometra are descriptive studies, most of which are case reports of one or a few cases. Against this background, there is currently no established diagnosis or treatment protocol. Therefore, future analytical and observational studies on genital tuberculosis presenting as pyometra are warranted.

## 4 Conclusion

*M. tuberculosis* should be considered a potential causative pathogen of pyometra, particularly in elderly or immunosuppressed patients.

## CRediT authorship contribution statement

**Kentaro Kasuga:** Writing – original draft, Data curation. **Miyako Kitazono:** Writing – review & editing. **Masaomi Maeda:** Writing – review & editing. **Taro Koba:** Writing – review & editing. **Shuichi Matsuda:** Writing – review & editing. **Mikio Takamori:** Writing – review & editing, Supervision.

## Funding

This research did not receive any specific grant from funding agencies in the public, commercial, or not-for-profit sectors.

## Declaration of competing interest

The authors declare that they have no known competing financial interests or personal relationships that could have appeared to influence the work reported in this paper.
